# Remodeling of Hepatic Glucose Metabolism in Response to Early Weaning in Piglets

**DOI:** 10.3390/ani14020190

**Published:** 2024-01-06

**Authors:** Chengbing Yu, Di Wang, Cheng Shen, Zhen Luo, Hongcai Zhang, Jing Zhang, Weina Xu, Jianxiong Xu

**Affiliations:** Shanghai Key Laboratory for Veterinary and Biotechnology, School of Agriculture and Biology, Shanghai Jiao Tong University, Shanghai 200240, China; yuchengbing@sjtu.edu.cn (C.Y.);

**Keywords:** early weaning, piglet, glucose metabolism, TCA cycle, liver, nutrition

## Abstract

**Simple Summary:**

In intensive farming, due to economic and management reasons, piglets have been progressively weaned earlier at 3 to 4 weeks of age. Early weaning usually leads to low feed intake and weight loss, which causes undernutrition and energy deficits. However, the effects of early weaning on hepatic glucose metabolism in piglets remain unclear. The objective of this study was to evaluate the dynamic changes in the hepatic glucose metabolism of 60 piglets at 14 days postweaning. We found that early weaning remodeled the content of metabolites related to glycometabolism and enhanced gluconeogenesis to produce more hepatic glucose. Further study showed that early weaning reduced the content of oxaloacetate and succinate, which suppressed ATP (adenosine triphosphate) production. Our findings enrich weaning stress theory and might provide a reference for dietary intervention.

**Abstract:**

This study aimed to investigate the dynamic changes in hepatic glucose metabolism in response to early weaning. A total of 60 piglets were randomly selected and weaned at 21 days old. Six piglets were slaughtered on the weaning day (d0) and at 1 (d1), 4 (d4), 7 (d7), and 14 (d14) days postweaning. The results illustrated that body weight significantly increased from d4 to d14 (*p* < 0.001). Serum glucose fell sharply after weaning and then remained at a low level from d1 to d14 (*p* < 0.001). Serum insulin decreased from d4 (*p* < 0.001), which caused hepatic glycogen to be broken down (*p* = 0.007). The glucose-6-phosphatase activity increased from d0 to d4 and then decreased from d4 to d14 (*p* = 0.039). The pyruvate carboxylase activity presented a significant sustained increase from d0 to d14 (*p* < 0.001). The succinate (*p* = 0.006) and oxaloacetate (*p* = 0.003) content on d4 was lower than that on d0. The succinate dehydrogenase activity (*p* = 0.008) and ATP (*p* = 0.016) production decreased significantly on d4 compared to that on d0. Taken together, these findings reveal the dynamic changes of metabolites and enzymes related to hepatic glycometabolism and the TCA (tricarboxylic acid) cycle in piglets after weaning. Our findings enrich weaning stress theory and might provide a reference for dietary intervention.

## 1. Introduction

Under natural conditions, weaning is a gradual process in pigs and is completed at 13 to 17 weeks of life, on average [1]. In intensive farming, due to economic and management reasons, piglets have been weaned progressively earlier at 3 to 4 weeks of age [2]. Early weaning is an abrupt event involving separation from sows and litter mates, transportation, dietary transition, and environment adaptation, which contribute significantly to weaning stress [3]. It has been reported that weaning stress results in high susceptibility to enteric disease, disturbed antioxidant capacity, poor growth performance, and increased mortality [4,5,6], causing huge economic losses in the pig industry.

Weaning stress is usually accompanied by low food intake due to the abrupt adaptation from the consumption of breast milk to a dry and solid diet (which is less digestible and palatable for the piglet), which can result in acute malnutrition and a reduced growth rate [7,8,9]. Glucose is an important source of energy and the liver plays a pivotal role in glucose homeostasis by controlling various pathways of glucose metabolism, including glycogenolysis, gluconeogenesis, and glycolysis [10,11]. In the fasted state, glucose from hepatic glycogenolysis and gluconeogenesis accounts for a substantial fraction of the total glucose production [12,13]. It is demonstrated that the gluconeogenesis rate in pigs declines after feeding and reaches the highest during energy deficit [14]. Evidence shows that weaning reduces blood glucose, which makes hepatic gluconeogenesis more important in weaning piglets [15]. The related intermediate content and enzyme activity can reflect the state of glucose metabolism [11]. It is reported that early weaning significantly affects glucose metabolism-related gene expression and enzyme activity in the liver [9,16]. However, further study is still required in terms of the importance of hepatic glucose metabolism in early weaned piglets.

The tricarboxylic acid (TCA) cycle is closely related to glycometabolism. The pyruvate derived from glucose metabolism enters the TCA cycle and is metabolized for energy. The TCA cycle not only serves as a main source of cellular energy, but also provides precursors for biosynthetic pathways. Some enzymes, including α-ketoglutarate dehydrogenase (α-KGDH), succinate dehydrogenase (SDH), and malate dehydrogenase (MDH), are essential in the TCA cycle, whose deficiency elicits profound metabolic disorders [17]. As metabolic signaling molecules, α-ketoglutaric acid has been found to decrease blood glucose and hepatic gluconeogenesis in mice with diet-induced obesity [18]. Circulating succinate can protect against diet-induced obesity and improve glucose tolerance via controlling the activation of adipose tissue thermogenesis [19]. In recent years, studies have found that intermediates derived from the TCA cycle can also act as nonmetabolic signaling mediators in proinflammatory and anti-inflammatory activity [20]. Some intermediates from the TCA cycle have been used as feed additives applied in the pig industry [21,22]. However, the effects of early weaning on the intermediates and enzymes in the TCA cycle remain unclear.

Therefore, the present study investigated the dynamic changes in the metabolites and enzymes related to hepatic glucose metabolism and the TCA cycle in weaning piglets. This study might provide a new perspective for understanding the effects of weaning stress on liver metabolism and contribute to the development of nutritional interventions.

## 2. Materials and Methods

### 2.1. Experimental Design and Feeding Management

A total of 60 piglets (Landrace × Yorkshire × Duroc) from 6 litters (10 of each) were randomly selected and weaned at 21 days old. Six piglets (1 piglet per litter, 3 males and 3 females) were randomly selected and slaughtered on the weaning day (d0) and at 1 (d1), 4 (d4), 7 (d7), and 14 (d14) days postweaning. The experimental diets were formulated according to the swine nutrition requirements recommended by the NRC (National Research Council) [23]. The dietary composition and nutrient levels are shown in Table 1. The piglets were housed in plastic-floored pens, where the temperature and humidity were controlled at around 25 °C and 60%, respectively. Each pen was equipped with a feeder and nipple drinker, and the piglets had ad libitum access to feed and water during the experimental period.

### 2.2. Sample Collection

At the selected time points postweaning, six piglets were randomly selected and euthanized via the intramuscular injection of 4% sodium pentobarbital solution (40 mg/kg body weight). All piglets were fasted for 12 h before sampling. Body weight was recorded on the day that the piglets were sacrificed. Blood samples were collected from the pigs’ precaval vein and then centrifuged (3000× *g*, 10 min, 4 °C) to obtain serum. Livers were rinsed thoroughly with ice-cold PBS to remove blood. The serum and liver samples were frozen immediately at −80 °C for further analysis.

### 2.3. Serum Glucose and Hepatic Glycogen Content

The serum glucose was detected according to the glucose oxidase/peroxidase method [24]. A total of 75 µL serum was added into the same volume of distilled water with sufficient mix. The mixture was placed into a boiling water bath for 10 min and then cooled to room temperature. The 20 µL supernatant was taken for analysis after centrifugation (8000× *g*, 10 min, 25 °C). The absorbance was read at a wavelength of 505 nm. The hepatic glycogen content was detected using the anthrone–sulfuric acid method [25]. Briefly, glycogen was extracted using an alkaline solution and then reacted with anthrone in the presence of concentrated sulfuric acid. The reaction mixture was centrifuged (8000× *g*, 10 min, 25 °C), and then the supernatant was taken for further analysis. The absorbance was read at a wavelength of 620 nm. A Blood Glucose Content Assay Kit and Glycogen Content Assay Kit were used according to the manufacturer’s instructions (Solarbio Life Science, Beijing, China). The absorbance was detected using a microplate reader (Synergy 2, BioTek, Winooski, VT, USA).

### 2.4. ATP Detection

The ATP content was measured with firefly luciferase according to the manufacturer’s instructions (Beyotime Biotechnology, Shanghai, China). The kits are based on the principle that firefly luciferase catalyzes luciferin to produce fluorescence, which requires ATP to provide energy. Briefly, the liver samples were homogenized and then the supernatant was taken for detection after being centrifuged (8000× *g*, 5 min, 4 °C). Black 96-well plates were used to minimize background fluorescence and light scattering. Working solution was added into the plates first to remove background ATP and then the samples were tested. The luminescence intensity was detected using a microplate reader (Synergy 2, BioTek, Winooski, VT, USA).

### 2.5. Determination of Enzyme Activity

The liver samples stored at −80 °C were used to measure the enzyme activity. Briefly, the liver samples were homogenized in PBS, and then the supernatant was taken for detection after being centrifuged (8000× *g*, 10 min, 4 °C). The enzyme activity of glucose-6-phosphatase (G6PC), phosphoenolpyruvate carboxykinase (PEPCK), pyruvate kinase (PK), phosphofructokinase (PFK), hexokinase (HK), α-ketoglutarate dehydrogenase (α-KGDH), and malate dehydrogenase (MDH) were measured using a microplate reader at 340 nm in 96-well UV microplates according to the kits’ instructions (Solarbio Life Science, Beijing, China). In particular, for the activity of pyruvate carboxylase (PC) and succinate dehydrogenase (SDH), the mitochondria were extracted via centrifugation (11,000× *g*, 15 min, 4 °C), and then disrupted using ultrasonication (SCIENTZ08-III, SCIENTZ, Ningbo, China). The main procedures were as follows: 12 cycles of ultrasonication for 5 s and an interval of 10 s at 20% power at 4 °C.

### 2.6. Enzyme-Linked Immunosorbent Assay (ELISA)

The serum samples were directly detected and the liver samples were homogenized in PBS. Then, the supernatant was taken for detection after centrifugation (4500× *g*, 10 min, 4 °C). Briefly, the samples and standard were added to plates pre-coated with antibodies, followed by incubation with horseradish-peroxidase-conjugated substrate at 37 °C for 60 min. The reaction mixture was removed and then washed thoroughly. The chromogen solutions A and B were added into each well. The mixture was incubated for 15 min at 37 °C away from light. After adding stop solution, the color of the reaction mixture turned yellow. The optical density was measured spectrophotometrically at a 450 nm wavelength. The concentrations were calculated according to the standard curve. The kits used for detecting serum insulin and glucagon were obtained from Gaining Biotech (Shanghai, China), and hepatic glycogen synthase 2 (GYS2) and glycogen phosphorylase (PYGL) were obtained from JYMbio (Wuhan, China).

### 2.7. Targeted Liver Metabolomics

Simply, the frozen samples (10 mg) were homogenized for 3 min with 10 beads and deionized water (20 µL). Then, 120 µL methanol was added to the mixture (including internal standard) for automated homogenization. The supernatant was collected using high-speed centrifugation (18,000× *g*, 4 °C, 15 min). The supernatant (20 µL), 3-nitrophenylhydrazine (200 mM, 20 µL), and EDC (120 mM, 20 µL) were sequentially added to each well of the 96-well plate, and then centrifuged (500× *g*, 30 °C, 60 min). The reaction mixture was diluted with 350 µL pre-chilled methanol. Then, 150 µL of the supernatant was taken for analysis after centrifugation (4000× *g*, 4 °C, 20 min). The analysis was performed by the Metabo-Profile R&D Laboratory (Shanghai, China) with UPLC-MS/MS (ACQUITY UPLC-Xevo TQ-S, Waters Corp., Milford, MA, USA).

### 2.8. Statistical Analysis

The normal distribution and homogeneity of variance were checked using the Shapiro–Wilk test and Levene’s test, respectively. If the conditions were met, one-way ANOVA and Tukey’s HSD post hoc tests were employed for multiple comparisons. If not, the Kruskal–Wallis test was used, followed by Dunn’s post hoc test. All data were presented as mean ± SEM and differences were considered statistically significant when *p* < 0.05 (* *p* < 0.05, ** *p* < 0.01, *** *p* < 0.01). Analyses were performed using SPSS 20.0 (IBM Corporation, Armonk, NY, USA). All figures were graphed using GraphPad Prism 9.0 (GraphPad Software, San Diego, CA, USA).

## 3. Results

### 3.1. Body Weight, Serum Biochemical, and Glycogen Metabolism Indicators

As shown in Table 2, body weight showed no obvious increase from d0 to d4 and continued to elevate from d4 to d14 (*p* < 0.001). In response to early weaning, serum glucose fell sharply after weaning and then remained at a low level from d1 to d14 (*p* < 0.001). The insulin secretion decreased significantly from d4 (*p* < 0.001) and remained at a low level until d14. The glucagon level showed no significant changes from d0 to d7 and then increased significantly on d14 (*p* < 0.001). To provide more glucose, hepatic glycogen was broken down and reached its lowest point on d4 (*p* = 0.007). The GYS2 content generally showed a rising trend, but no statistical difference was observed during the experimental period (*p* = 0.322). The PYGL content showed a significant increase on d1 compared to that on d0 (*p* = 0.049).

### 3.2. Weaning Remodeled Metabolites Related to Glycometabolism

**Figure 1:**
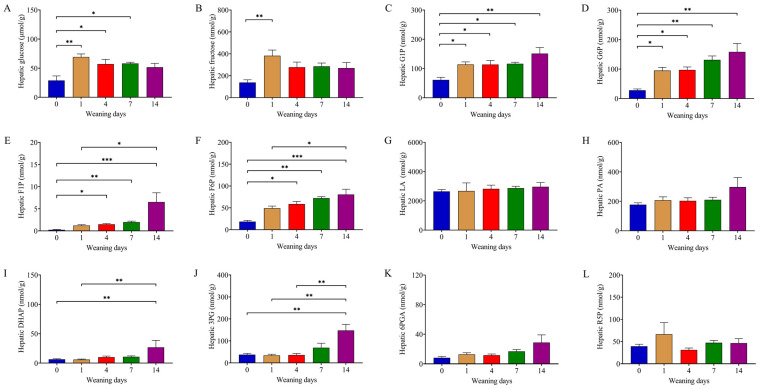
Developmental changes in metabolites related to glycometabolism. (**A**–**L**) Hepatic glucose, fructose, G1P, G6P, F1P, F6P, LA, PA, DHAP, 3PG, 6PGA, and R5P content detected using UPLC-MS/MS. Glucose and fructose were analyzed using one-way ANOVA followed by Tukey’s HSD post hoc test, and the others were analyzed via Kruskal–Wallis test followed by Dunn’s post hoc test. Data presented as mean ± SEM (n = 6). The numbers 0, 1, 4, 7, and 14 represent the days after weaning in 21-day-old piglets. * *p* < 0.05, ** *p* < 0.01, *** *p* < 0.01. Glucose: *D*-glucose, fructose: *D*-fructose, G1P: glucose-1-phosphate, G6P: glucose-6-phosphate, F1P: fructose-1-phosphate, F6P: fructose-6-phosphate, LA: *L*-lactic acid, PA: pyruvic acid, DHPA: dihydroxyacetone phosphate, 3PG: 3-phosphoglyceric acid, 6PGA: 6-phosphogluconic acid, R5P: *D*-ribulose-5-phosphate.

Considering the importance of glucose in maintaining normal physiological functions, the related metabolites in the liver were further detected. The content of glucose (*p* = 0.003) and fructose (*p* = 0.010) maintained a high level during the experimental period and both increased significantly on d1 compared to d0 (Figure 1A,B). The glucose-1-phosphate (G1P, *p* = 0.013) showed a similar trend with glucose-6-phosphate (G6P, *p* = 0.002), whose content on d0 was significantly lower than the other four time points (Figure 1C,D). The content of fructose-1-phosphate (F1P, *p* < 0.001) and fructose-6-phosphate (F6P, *p* < 0.001) on d4, d7, and d14 were significantly higher than that on d0 (Figure 1E,F). There was no significant difference in lactic acid (LA, *p* = 0.456) or pyruvic acid (PA, *p* = 0.348) content in the statistics from d0 to d14 (Figure 1G,H). The hepatic content of dihydroxyacetone phosphate (DHAP, *p* = 0.017) on d14 was significantly higher than that on d0 and d1 (Figure 1I). The hepatic 3-phosphoglyceric acid (3PG, *p* = 0.007) content on d14 was significantly higher than that on d0, d1, and d4 (Figure 1J). There was no statistically significant difference in hepatic content of 6-phosphogluconic acid (6PGA, *p* = 0.136) and ribulose-5-phosphate (R5P, *p* = 0.206) during the 14 days postweaning (Figure 1K,L).

### 3.3. Weaning Enhanced Activity of Enzymes in Gluconeogenesis

**Figure 2:**
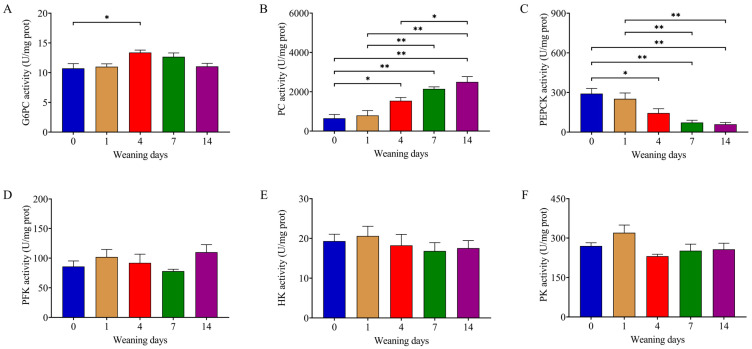
Enzyme activity in glycolysis and gluconeogenesis. (**A**–**F**) Activity of G6PC, PC, PEPCK, PFK, HK, and PK. Data were analyzed using one-way ANOVA followed by Tukey’s HSD post hoc test and presented as mean ± SEM (n = 6). The numbers 0, 1, 4, 7, and 14 represent the days after weaning in 21-day-old piglets. * *p* < 0.05, ** *p* < 0.01. G6PC: glucose-6-phosphatase, PC: pyruvate carboxylase, PEPCK: phosphoenolpyruvate carboxykinase, PFK: phosphofructokinase, HK: hexokinase, PK: pyruvate kinase.

The liver is the major site of glycolysis and gluconeogenesis. Glucose-6-phosphatase (G6PC), phosphoenolpyruvate carboxykinase (PEPCK), and pyruvate carboxylase (PC) are the key rate-limiting enzymes in gluconeogenesis. Hexokinase (HK), pyruvate kinase (PK), and phosphofructokinase (PFK) are the key rate-limiting enzymes in glycolysis. The activity of G6PC (*p* = 0.039) significantly increased on d4 compared to that on d0 (Figure 2A). The activity of PC (*p* < 0.001) and PEPCK (*p* < 0.001) generally showed an uptrend and downtrend after weaning, respectively (Figure 2B,C). No significant difference in PFK (*p* = 0.312), HK (*p* = 0.770), or PK (*p* = 0.068) activity was observed between the different time points after weaning (Figure 2D–F).

### 3.4. Weaning Decreased Content of Succinate in the TCA Cycle

**Figure 3:**
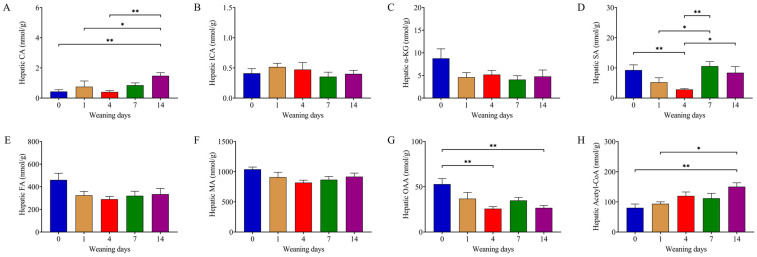
Intermediate metabolites in the TCA cycle. (**A**–**H**) Hepatic CA, ICA, α-KG, SA, FA, MA, OAA, and acetyl-CoA content detected using UPLC-MS/MS. ICA, FA, MA, OAA, and acetyl-CoA were analyzed using one-way ANOVA followed by Tukey’s HSD post hoc test and the others were analyzed using the Kruskal–Wallis test followed by Dunn’s post hoc test. Data presented as mean ± SEM (n = 6). The numbers 0, 1, 4, 7, and 14 represent the days after weaning in 21-day-old piglets. * *p* < 0.05, ** *p* < 0.01. CA: citric acid, ICA: isocitric acid, α-KG: alpha-ketoglutarate, SA: succinic acid, FA: fumaric acid, MA: *L*-malic acid, OAA: oxalacetic acid, acetyl-CoA: acetyl coenzyme A.

The TCA cycle is a metabolic pathway used to generate energy and intermediates for biosynthetic pathways. As the first intermediate of the TCA cycle, the content of citric acid (CA, *p* = 0.010) significantly increased on d14 compared to that on d0, d1, and d4 (Figure 3A). The content of succinic acid (SA, *p* = 0.006) on d4 was significantly lower than on d0, d7, and d14 (Figure 3D). D4 and d14 showed a lower oxalacetic acid (OAA, *p* = 0.003) content compared with d0 (Figure 3G). Overall, the content of acetyl-CoA (*p* = 0.005) exhibited a rising tendency during the experimental period, and d14 showed a significantly higher acetyl-CoA content than d1 and d0 (Figure 3H). There was no difference in isocitric acid (ICA, *p* = 0.652), α-ketoglutarate (α-KG, *p* = 0.412), fumaric acid (FA, *p* = 0.084), or malic acid (MA, *p* = 0.104) content in the statistics during the experiment (Figure 3B,C,E,F).

### 3.5. Weaning Reduced SDH Activity and ATP Production

**Figure 4:**
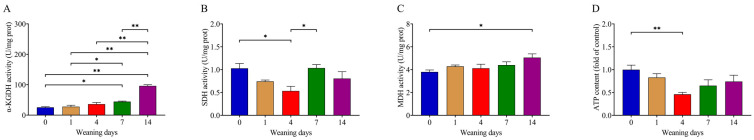
ATP generation and enzyme activity in the TCA cycle. (**A**–**C**) Activity of α-KGDH, MDH, and SDH related to the TCA cycle, (**D**) ATP generation. Data were analyzed using one-way ANOVA followed by Tukey’s HSD post hoc test and presented as mean ± SEM (n = 6). The numbers 0, 1, 4, 7, and 14 represent the days after weaning in 21-day-old piglets. * *p* < 0.05, ** *p* < 0.01. ATP: adenosine triphosphate, α-KGDH: α-ketoglutarate dehydrogenase, MDH: malate dehydrogenase, SDH: succinate dehydrogenase.

The enzymes related to the TCA cycle play a pivotal role in maintaining the cycle’s function. The activity of α-ketoglutarate dehydrogenase (α-KGDH, *p* < 0.001) on d0 and d1 was lower than that on d7 and d14 (Figure 4A). There was a significant difference in malate dehydrogenase (MDH, *p* = 0.035) activity between d14 and d0 (Figure 4C). The activity of succinate dehydrogenase (SDH, *p* = 0.008) showed a similar trend as the SA content, which reduced to the minimum on d4 and then increased to the maximum on d7 (Figure 4B). The adenosine triphosphate (ATP, *p* = 0.016) production first decreased from d0 to d4 and then increased from d4 to d14, with statistical significance between d0 and d4 being observed (Figure 4D).

## 4. Discussion

Weaning stress is a bottleneck problem that restricts the healthy development of pigs in the pig industry. Previous studies reported that early weaning causes disorders related to redox homeostasis and triggers cell apoptosis, which indicates liver function injury in piglets [26,27]. A comparative analysis revealed that the hepatic glucogenesis capacity is gradually weakened in suckling piglets and glucose catabolism is significantly suppressed by early weaning [16]. The liver plays a crucial role in glucose homeostasis by regulating hepatic glycogen storage and glucose production. When blood glucose falls, hepatic glycogen breaks down to provide glucose. In the fasted state, insulin secretion ceases and glucagon secretion is enhanced, leading to the inhibition of GYS2 and the activation of PYGL [28,29]. Early weaning usually causes an energy deficiency, which makes glucose metabolism particularly important for piglets [9]. In this study, we found that even hepatic glucose production doubled after weaning, the blood glucose still decreased sharply and then maintained at low levels though. The reason for this may be that the liver provides glucose to fuel obligate glucose-consuming cell types, such as neurons, red blood cells, and renal medullary cells, as a priority when glucose is in shortage [30,31]. To satisfy the demand for glucose, the circulating insulin declined from d1, which made the hepatic glycogen break down. These data indicate that the demand for glucose increases in piglets, especially at the early stage after weaning. 

The liver produces glucose mainly via two biological processes: glycogenolysis and gluconeogenesis. In the fasted state, glucose from hepatic glycogenolysis and gluconeogenesis accounts for a substantial fraction of the total glucose production [12,13]. In this study, glucose and fructose, as well as their metabolites G1P, G6P, F1P, and F6P, generally showed an increasing trend after weaning, indicating that the carbohydrate metabolism was more active. G6PC, PC, and PEPCK are the key rate-limiting enzymes in gluconeogenesis. G6PC catalyzes G6P to form glucose at the terminal step of gluconeogenesis and glycogenolysis, and PC converts PA to OAA [32]. Here, we found that G6PC and PC both showed a considerable increase on d4 compared to on d0, suggesting that weaning enhances the gluconeogenesis process. Low hepatic PEPCK expression can lead to hypoglycemia, especially during fasting [33]. PEPCK activity was found to gradually decrease after weaning in this study, which was consistent with a previous study [16]. No statistically significant changes in HK, PK, or PFK activity were detected, which suggests that early weaning has a slight impact on glycolysis during the first 2 weeks postweaning. The reason for this may be that glycolysis is less efficient than oxidative phosphorylation in ATP yield; glucose is used to sustain normal brain function and cardiac rhythm rather than generating energy when glucose is undersupplied [30,31]. 

The TCA cycle is closely related to glycometabolism. Through the breakdown of carbohydrates through glycolysis, acetyl-CoA enters the TCA cycle by combining with OAA to form CA. The TCA cycle is an important metabolic pathway that connects carbohydrate, lipid, and protein metabolism and produces usable chemical energy in the form of ATP. In recent years, the metabolites and derivatives from the TCA cycle have drawn extensive attention for their metabolic and nonmetabolic signaling functions [34,35]. SA plays a pivotal role in metabolism as the only direct link between the TCA cycle and the mitochondrial respiratory chain. SDH, also named respiratory complex II, catalyzes the oxidation of SA to FA. It is reported that SA accumulation in ischemia and anoxia causes inflammation by regulating succinate receptor and ROS production [36,37,38,39]. Research shows that SA stimulates insulin secretion and proinsulin biosynthesis [39]. Microbiota-produced succinate can improve glucose homeostasis via intestinal gluconeogenesis [40]. Dietary SA stimulates beige adipogenesis by promoting mitochondrial complex II activity, thus combating obesity [41]. These studies indicate that SA plays an important role in the inflammatory response and energy metabolism. In this study, the content of SA and ATP, together with SDH activity, were found to be lower on d4 than on d0. These data suggest that early weaning reduces ATP generation, possibly by decreasing the SA content. The metabolites derived from the TCA cycle have been used as feed additives in animal husbandry for enhancing animal health and growth performance [21,42,43,44]. It is reported that dietary SA supplementation protects the integrity of the small intestinal epithelium in growing pigs [21]. Dietary SA affects intestinal bile acid transport and promotes hepatic bile acid synthesis and secretion in pigs [45]. The SA accumulation induced by early weaning increases fluid secretion in the epithelium and the immune response in macrophages, which causes severe diarrhea [46]. Of note, these studies indicate that SA supplementation may have different functions in pigs at different growth periods or under different physiological conditions. The liver is a crucial metabolic organ, but more research is required on the effects of SA on the liver in weaning piglets. Our findings provide a reference for the use of metabolites (especially SA) from the TCA cycle as feed additives in weaning piglets. 

In general, early weaning influences the content of metabolites and the activity of enzymes related to the TCA cycle, which results in a reduction in ATP generation. To maintain the glucose supply, gluconeogenesis and glycogenolysis are enhanced. Obviously, early weaning has a huge impact on the hepatic energy metabolism of piglets, but the mechanism behind this is still unclear. Moreover, the hepatic content of succinate significantly decreases after weaning, whose biological significance is worth in-depth study considering its regulatory roles on inflammation and metabolism in human diseases. Our study provides a reference for further research into animal and human health.

## 5. Conclusions

Taken together, early weaning remodeled the content of metabolites and the activity of enzymes related to the TCA cycle, which led to a decline in ATP generation and resulted in an energy deficit. In response to early weaning, the gluconeogenesis and glycogenolysis pathways were enhanced to satisfy the body’s demand for glucose. However, the influencing mechanism of early weaning on hepatic energy metabolism in piglets needs further study. Our findings enrich weaning stress theory and provide a reference for dietary intervention.

## Figures and Tables

**Table 1:** Dietary composition and nutrient level (%, as-fed basis).

Item	Content
Ingredients	
Corn	33.00
Extruded corn	22.00
Fermented soybean meal	11.20
Extruded soybean	8.00
Soybean meal	9.40
Fish meal	4.00
Whey powder	6.00
Soybean oil	1.40
White granulated sugar	1.35
Choline chloride	0.20
CaHPO_4_	1.20
Limestone	0.40
NaCl	0.30
*L*-Lys	0.33
*DL*-Met	0.12
*L*-Thr	0.10
Premix ^a^	1.00
Total	100.00
Calculated nutrient level	
Metabolizable energy (MJ/kg)	14.62
Crude protein	20.50
Crude fat	4.37
Crude fiber	2.34
Crude ash	5.80
Lys	1.17
Met + Cys	0.69
Thr	0.73
Try	0.19
Calcium	0.60
Total phosphorus	0.60

^a^ Premix provided the following per kilogram of diet: vitamin A, 3500 IU; vitamin C, 100 mg; vitamin D3, 350 IU; vitamin E, 30 IU; vitamin K, 0.8 mg; vitamin B1, 2.0 mg; vitamin B2, 6.0 mg; vitamin B3, 12.5 mg; vitamin B6, 3.0 mg; vitamin B12, 0.03 mg; choline chloride, 800 mg; d-pantothenic acid, 15.0 mg; folic acid, 0.45 mg; nicotinic acid, 22.5 mg; biotin, 0.08 mg; Cu (CuSO_4_·5H_2_O), 9.0 mg; Fe (FeSO_4_·H_2_O), 150 mg; Zn (ZnSO_4_·H_2_O), 150 mg; Mn (MnSO_4_·5H_2_O), 50 mg; I (KI) 0.25 mg; Se (Na_2_SeO_3_·H_2_O), 0.45 mg.

**Table 2:** Body weight, serum biochemical, and glycogen metabolism indicators.

Item	D0	D1	D4	D7	D14	*p*-Value
Body weight, kg	7.05 ± 0.16 ^bc^	6.58 ± 0.14 ^c^	6.62 ± 0.09 ^c^	7.34 ± 0.16 ^b^	9.08 ± 0.15 ^a^	<0.001
Serum glucose, mmol/L	5.95 ± 0.46 ^a^	4.41 ± 0.12 ^b^	4.15 ± 0.33 ^b^	3.84 ± 0.33 ^b^	3.82 ± 0.26 ^b^	<0.001
Serum insulin, µIU/mL	4.45 ± 0.16 ^a^	5.04 ± 0.31 ^a^	3.37 ± 0.17 ^b^	3.21 ± 0.31 ^b^	3.17 ± 0.10 ^b^	<0.001
Serum glucagon, pg/mL	310.91 ± 7.08 ^b^	327.57 ± 4.20 ^b^	315.69 ± 6.09 ^b^	314.95 ± 3.93 ^b^	360.42 ± 8.18 ^a^	<0.001
Hepatic glycogen, mg/g	40.49 ± 9.17 ^a^	18.38 ± 2.16 ^ab^	15.13 ± 2.87 ^b^	23.71 ± 3.17 ^ab^	42.25 ± 8.31 ^a^	0.007
Hepatic GYS2, pg/mg prot	16.84 ± 0.58	20.40 ± 1.50	19.40 ± 1.13	20.09 ± 1.34	17.63 ± 2.05	0.322
Hepatic PYGL, pg/mg prot	30.32 ± 1.73 ^b^	42.19 ± 3.37 ^a^	39.65 ± 1.19 ^ab^	41.28 ± 1.94 ^ab^	39.23 ± 4.58 ^ab^	0.049

Data presented as mean ± SEM (n = 6). All data were analyzed using one-way ANOVA followed by Tukey’s HSD post hoc test. Values in the same row with different letters (a, b, c) differed significantly (*p* < 0.05). GYS2: glycogen synthase 2, PYGL: glycogen phosphorylase.

## Data Availability

The data described in the manuscript will be made available upon request. Data are contained within the article.

## References

[1] Nielsen S., Alvarez J., Bicout D., Calistri P., Canali E., Drewe J., Garin-Bastuji B., Rojas J., Schmidt G., Herskin M. (2022). Welfare of pigs on farm. EFSA J..

[2] Edwards S., Turpin D.L., Pluske J. (2020). Weaning age and its long-term influence on health and performance. The Suckling and Weaned Piglet.

[3] Tang X., Xiong K., Fang R., Li M. (2022). Weaning stress and intestinal health of piglets: A review. Front. Immunol..

[4] Yin J., Wu M.M., Xiao H., Ren W.K., Duan J.L., Yang G., Li T.J., Yin Y.L. (2014). Development of an antioxidant system after early weaning in piglets. J. Anim. Sci..

[5] Hu J., Ma L., Nie Y., Chen J., Zheng W., Wang X., Xie C., Zheng Z., Wang Z., Yang T. (2018). A Microbiota-Derived Bacteriocin Targets the Host to Confer Diarrhea Resistance in Early-Weaned Piglets. Cell Host Microbe.

[6] Faccin J., Laskoski F., Hernig L., Kummer R., Lima G., Orlando U., Gonçalves M., Mellagi A.P., Ulguim R., Bortolozzo F. (2020). Impact of increasing weaning age on pig performance and belly nosing prevalence in a commercial multisite production system. J. Anim. Sci..

[7] Campbell J., Crenshaw J., Polo J. (2013). The biological stress of early weaned piglets. J. Anim. Sci. Biotechnol..

[8] Mota Rojas D., Roldán Santiago P., Pérez Pedraza E., Martínez Rodríguez R., Henrández Trujillo E., Trujillo Ortega M.E. (2014). Stress factors in weaned piglet. Vet. Mex..

[9] Le Dividich J., Sève B. (2000). Effects of underfeeding during the weaning period on growth, metabolism, and hormonal adjustments in the piglet. Domest. Anim. Endocrinol..

[10] Rui L. (2014). Energy Metabolism in the Liver. Compr. Physiol..

[11] Han H.S., Kang G., Kim J.S., Choi B.H., Koo S.H. (2016). Regulation of glucose metabolism from a liver-centric perspective. Exp. Mol. Med..

[12] Ekberg K., Landau B.R., Wajngot A., Chandramouli V., Efendic S., Brunengraber H., Wahren J. (1999). Contributions by kidney and liver to glucose production in the postabsorptive state and after 60 h of fasting. Diabetes.

[13] Rothman D., Magnusson I., Katz L., Shulman R., Shulman G. (1991). Quantitation of Hepatic Glycogenolysis And Gluconeogenesis in Fasting Humans with ^13^C NMR. Science.

[14] Duee P.-H., Pegorier J.-P., Darcy-Vrillon B., Girard J. (1996). Glucose and Fatty Acid Metabolism in the Newborn Pig. Advances in Swine in Biomedical Research.

[15] Jarratt L., James S.E., Kirkwood R.N., Nowland T.L. (2023). Effects of Caffeine and Glucose Supplementation at Birth on Piglet Pre-Weaning Growth, Thermoregulation, and Survival. Animals.

[16] Xie C., Wang Q., Wang J., Tan B., Fan Z., Deng Z., Wu X., Yin Y. (2016). Developmental changes in hepatic glucose metabolism in a newborn piglet model: A comparative analysis for suckling period and early weaning period. Biochem. Biophys. Res. Commun..

[17] Kang W., Suzuki M., Saito T., Miyado K. (2021). Emerging Role of TCA Cycle-Related Enzymes in Human Diseases. Int. J. Mol. Sci..

[18] Yuan Y., Zhu C., Wang Y., Sun J., Feng J., Ma Z., Li P., Peng W., Yin C., Xu G. (2022). α-Ketoglutaric acid ameliorates hyperglycemia in diabetes by inhibiting hepatic gluconeogenesis via serpina1e signaling. Sci. Adv..

[19] Mills E., Pierce K., Jedrychowski M., Garrity R., Winther S., Vidoni S., Yoneshiro T., Spinelli J.B., Lu G., Kazak L. (2018). Accumulation of succinate controls activation of adipose tissue thermogenesis. Nature.

[20] Martínez-Reyes I., Chandel N. (2020). Mitochondrial TCA cycle metabolites control physiology and disease. Nat. Commun..

[21] Li X., Mao M., Zhang Y., Yu K., Zhu W. (2019). Succinate Modulates Intestinal Barrier Function and Inflammation Response in Pigs. Biomolecules.

[22] Wang L., Yi D., Hou Y., Ding B., Li K., Li B., Zhu H., Liu Y., Wu G. (2016). Dietary Supplementation with -Ketoglutarate Activates mTOR Signaling and Enhances Energy Status in Skeletal Muscle of Lipopolysaccharide-Challenged Piglets. J. Nutr..

[23] NRC (2012). Nutrient Requirements of Swine: Eleventh Revised Edition.

[24] Kabasakalian P., Kalliney S., Westcott A. (1974). Enzymatic Blood Glucose Determination by Colorimetry of N-Diethylaniline-4-Aminoantipyrine. Clin. Chem..

[25] Roe J., Dailey R. (1966). Determination of glycogen with the anthrone reagent. Anal. Biochem..

[26] Luo Z., Zhu W., Guo Q., Luo W., Zhang J., Xu W., Xu J. (2016). Weaning Induced Hepatic Oxidative Stress, Apoptosis, and Aminotransferases through MAPK Signaling Pathways in Piglets. Oxid. Med. Cell. Longev..

[27] Yu L., Li H., Peng Z., Ge Y., Liu J., Wang T., Wang H., Dong L. (2021). Early Weaning Affects Liver Antioxidant Function in Piglets. Animals.

[28] Campbell J.E., Newgard C.B. (2021). Mechanisms controlling pancreatic islet cell function in insulin secretion. Nat. Rev. Mol. Cell Biol..

[29] Zhang L., Yao W., Xia J., Wang T., Huang F. (2019). Glucagon-Induced Acetylation of Energy-Sensing Factors in Control of Hepatic Metabolism. Int. J. Mol. Sci..

[30] Amiel S.A. (2021). The consequences of hypoglycaemia. Diabetologia.

[31] Petersen M., Vatner D., Shulman G. (2017). Regulation of hepatic glucose metabolism in health and disease. Nat. Rev. Endocrinol..

[32] Cappel D.A., Deja S., Duarte J.A.G., Kucejova B., Iñigo M., Fletcher J.A., Fu X., Berglund E.D., Liu T., Elmquist J.K. (2019). Pyruvate-Carboxylase-Mediated Anaplerosis Promotes Antioxidant Capacity by Sustaining TCA Cycle and Redox Metabolism in Liver. Cell Metab..

[33] Beale E.G., Harvey B.J., Forest C. (2007). PCK1 and PCK2 as candidate diabetes and obesity genes. Cell Biochem. Biophys..

[34] Eniafe J., Jiang S. (2021). The functional roles of TCA cycle metabolites in cancer. Oncogene.

[35] Murphy M.P., O’Neill L.A.J. (2018). Krebs Cycle Reimagined: The Emerging Roles of Succinate and Itaconate as Signal Transducers. Cell.

[36] Mills E., Harmon C., Jedrychowski M., Xiao H., Garrity R., Tran N., Bradshaw G., Fu A., Szpyt J., Reddy A. (2021). UCP1 governs liver extracellular succinate and inflammatory pathogenesis. Nat. Metab..

[37] Hass D., Bisbach C., Robbings B., Sadilek M., Sweet I., Hurley J. (2022). Succinate metabolism in the retinal pigment epithelium uncouples respiration from ATP synthesis. Cell Rep..

[38] Mills E., Kelly B., Logan A., Costa A., Varma M.M., Bryant C., Tourlomousis P., Däbritz J.H., Gottlieb E., Latorre I. (2016). Succinate Dehydrogenase Supports Metabolic Repurposing of Mitochondria to Drive Inflammatory Macrophages. Cell.

[39] Attali V., Parnes M., Ariav Y., Cerasi E., Kaiser N., Leibowitz G. (2006). Regulation of Insulin Secretion and Proinsulin Biosynthesis by Succinate. Endocrinology.

[40] De Vadder F., Kovatcheva-Datchary P., Zitoun C., Duchampt A., Bäckhed F., Mithieux G. (2016). Microbiota-Produced Succinate Improves Glucose Homeostasis via Intestinal Gluconeogenesis. Cell Metab..

[41] Liu K., Lin L., Li Q., Xue Y., Zheng F., Wang G., Zheng C., Du L., Hu M., Huang Y. (2020). Scd1 controls de novo beige fat biogenesis through succinate-dependent regulation of mitochondrial complex II. Proc. Natl. Acad. Sci. USA.

[42] Chen J.S., Wu F., Yang H.S., Li F.N., Jiang Q., Liu S.J., Kang B.J., Li S., Adebowale T.O., Huang N. (2017). Growth performance, nitrogen balance, and metabolism of calcium and phosphorus in growing pigs fed diets supplemented with alpha-ketoglutarate. Anim. Feed Sci. Technol..

[43] Meng M., Zhao X., Huo R., Li X., Chang G., Shen X. (2023). Disodium Fumarate Alleviates Endoplasmic Reticulum Stress, Mitochondrial Damage, and Oxidative Stress Induced by the High-Concentrate Diet in the Mammary Gland Tissue of Hu Sheep. Antioxidants.

[44] Qiu K., He W., Zhang H., Wang J., Qi G., Guo N., Zhang X., Wu S. (2022). Bio-Fermented Malic Acid Facilitates the Production of High-Quality Chicken via Enhancing Muscle Antioxidant Capacity of Broilers. Antioxidants.

[45] Li X., Ren Y., Huang G., Zhang R., Zhang Y., Zhu W., Yu K. (2022). Succinate communicates pro-inflammatory signals to the host and regulates bile acid enterohepatic metabolism in a pig model. Food Funct..

[46] Zhou X., Liu Y., Xiong X., Chen J., Tang W., He L., Zhang Z., Yin Y., Li F. (2022). Intestinal accumulation of microbiota-produced succinate caused by loss of microRNAs leads to diarrhea in weanling piglets. Gut Microbes.

